# Online monitored characterization of *Phocaeicola vulgatus* for organic acid production using anaerobic microtiter plate cultivations

**DOI:** 10.1002/btpr.3526

**Published:** 2024-12-20

**Authors:** Laura Keitel, Benjamin Schick, Gino Pohen, Stanislav Yordanov, Jochen Büchs

**Affiliations:** ^1^ RWTH Aachen University Chair of Biochemical Engineering (AVT.BioVT) Aachen Germany

**Keywords:** anaerobic fermentation, BioLector, gut bacteria, online monitoring, *Phocaeicola (Bacteroides) vulgatus*, short chain fatty acids (SCFA)

## Abstract

*Phocaeicola vulgatus* (formerly *Bacteroides vulgatus*), an anaerobic gut bacterium, produces several organic acids. Research on *P. vulgatus* is still in its infancy. However, a detailed understanding of *P. vulgatus* growth and metabolism is essential for its assessment as an organic acid producer. Media variations, including different initial glucose and NH_4_Cl concentrations and osmolalities, are significant means to yield higher organic acid titers. Furthermore, examining different nitrogen and carbon sources is important to evaluate the potential of *P. vulgatus* for growth on renewable resources. Cultivations were performed in an in‐house built device for anaerobic online‐monitoring of fluorescence and scattered light in microtiter plates. Results revealed that the highest organic acid concentrations were reached while using galactose, glucose, or xylose as a carbon source, high osmolalities, and 0.25 g L^−1^ NH_4_Cl. In addition, the organic acid composition changed with changing carbon and nitrogen sources. *P. vulgatus* was successfully further characterized, thereby contributing to a faster characterization of other anaerobic strains and paving the way for anaerobic organic acid production.

## 1 INTRODUCTION

The human intestine is a unique organ constantly exposed to different dietary‐, microbiota‐, and host‐derived influences. These influences lead to different physical and chemical conditions, such as acidity, temperature or osmolality, and environmental gradients along the intestine.^1^ It is also the habitat of the largest bacterial population in the body, with approximately 10^2^, ^3^ organisms per milliliter of colonic contents.^4^, ^5^ Gut bacteria support the maturation of the immune system.^6^ A developed intestine prevents its colonization by harmful bacteria and supports human metabolism by breaking down indigestible polysaccharides into nutrients, vitamins, co‐factors, amino acids, and short‐chain fatty acids (SCFAs).^5^, ^7^, ^9^ The phylum *Bacteroidota* dominates in the human intestine^4^, ^9^ and can produce high amounts of organic acids.^10^, ^11^, ^12^ *Phocaeicola vulgatus*, first identified as *Bacteroides vulgatus*,^12^ is one of the most abundant strains in human feces^8^ and has the potential to be genetically modified.^13^, ^14^ In addition, various studies show that *Bacteroidota* can synthesize antibiotic and bioactive components^3^, ^15^ and are applied as probiotics.^16^ However, *P. vulgatus* has not yet been used in biotechnological processes.^13^ Understanding its carbon metabolism is essential for using *P. vulgatus* for biotechnological applications.

The genetically related genus *Bacteroides* produces the organic acids acetate, propionate, succinate, lactate, formate, and the gasses CO_2_ and H_2_ through anaerobic respiration.^5^ During anaerobic respiration, they take advantage of the high CO_2_ levels in the gut and reduce fumarate to succinate.^5^ In addition, *P. vulgatus* forms lactate by reducing pyruvate via lactate dehydrogenase.^13^ *Prevotella copri*, another *P. vulgatus*‐related strain, can convert pyruvate to formate, CO_2_, Fd_red_ (possible site of H_2_ formation), and acetyl‐CoA.^17^ The acetyl‐CoA is then converted to acetate. Another significant component of the anaerobic respiratory chain is the membrane‐bound Na^+^ pump, an essential means of survival in the sodium‐rich gut ecosystem.^18^

Besides the carbon metabolism, the nitrogen metabolism plays a vital role in *P. vulgatus* survival.^5^ Many *Bacteroides* strains have a glutamate dehydrogenase to fix ammonia.^19^ Varel and Bryant^20^ have shown that organic nitrogen sources, such as amino acids, could not replace ammonia for several *Bacteroides* strains, including *P. vulgatus*. *Bacteroides* strains can also use alternative pathways to produce certain amino acids from SCFAs,^21^ supporting this hypothesis.

*Bacteroides* strains were described as efficient polysaccharide degraders.^8^ Complex carbohydrates represent their primary carbon source, as simple sugars are absorbed in the small intestine or consumed by other intestinal bacteria.^22^ In the human intestine, most polysaccharides are provided by the diet, for example, starch or plant cell walls consisting of pectin, hemicelluloses, and celluloses.^22^, ^23^ Pectin, for example, is then degraded by the intestinal bacteria with the aid of esterases to polygalacturonic acid and, further, galacturonic acid.^24^ In addition to plant polysaccharides, sugar alcohols such as sorbitol and glycerol^25^, ^26^ or prebiotics like inulin^27^ also find their way into the large intestine via the diet. Although *Bacteroides* are common and relevant to human gut health, they have not been well studied regarding their polysaccharide degradation abilities. Genomic screening of *P. vulgatus* revealed that the strain should not degrade levan and inulin.^28^ However, experiments have shown that *P. vulgatus* can efficiently degrade starch^28^, ^29^ and pectin.^30^, ^31^, ^32^

As previously described, one of the major product groups of *P. vulgatus* are SCFAs: acetate, propionate, succinate, and formate. Lactate, a short‐chain hydroxy fatty acid, described in this work as SCFA, is another product of *P. vulgatus*. SCFAs are metabolized by other bacteria^9^, ^33^ or serve the human host for various purposes, such as signal molecules or energy substrates.^34^, ^35^ SCFAs are also crucial for intestinal bacteria, to regulate the production of redox equivalents in the intestine.^36^ Furthermore, they are also used in the chemical industry and are currently produced primarily based on fossil fuels. Succinate in particular is expensive to produce and there is as yet no biotechnological production process in use.^37^ Therefore, sustainable production based on renewable raw materials is desired. *P. vulgatus* is a promising platform organism for the biotechnological synthesis of SCFAs, as it can use a variety of carbon sources as substrates, for example, from organic waste streams from agriculture, forestry and the food industry. Increasing SCFA production could be reached by cultivating *P. vulgatus* in an anaerobic mixed culture using these organic waste streams as a substrate.^38^, ^39^, ^40^, ^41^ As *P. vulgatus* exists naturally in mixed cultures, this form of cultivation is advantageous for its growth rate and cross‐feeding is performed, as shown by Kattel et.^42^ After *P. vulgatus* is characterized in axenic culture, which is the goal of this study, mixed culture cultivations could be an important next step to enhance the SCFA production.

Although interest in anaerobic bacteria for biotechnological processes is growing, little research has been done on *P. vulgatus* regarding biotechnological potential and characterization in axenic culture.^13^ This study aims to advance the characterization of *P. vulgatus* on small scale under anaerobic conditions, screening the influence of different media components for growth and the potential of *P. vulgatus* as an organic acid producer. In addition, alternative substrates, such as carbon and nitrogen sources, were tested to explore the potential of SCFA production with *P. vulgatus* based on renewable resources. For this purpose, shaken bioreactors on a microtiter plate (MTP) scale with online monitoring of scattered light, riboflavin, and nicotinamide adenine dinucleotide (NADH) fluorescence intensity were used, as the increased number of parameters to be investigated requires high throughput. This study's results will help to characterize *P. vulgatus* and assess the potential of *P. vulgatus* for biotechnological applications. It will further show the applicability of a shaken bioreactor on MTP scale with online monitoring for *P. vulgatus*.

## 2 MATERIALS AND METHODS

### 2.1 Strain and media

An axenic sample of the strain *Phocaeicola vulgatus* DSM 1447, obtained from the German Collection of Microorganisms and Cell Cultures (DSMZ, Brunswick, Germany), was kindly provided by the research group of Prof. Deppenmeier (Rheinische Friedrich‐Wilhelms‐Universität, Bonn, Germany). Initial cultivation was carried out in Brain heart infusion medium (BHI) acquired from BD Difco™ (Thermo Fisher, Waltham, USA). Brain heart infusion medium powder contained: 7.7 g L^−1^ calf brain, 9.8 g L^−1^ beef heart, 10 g L^−1^ protease peptone, 2 g L^−1^ dextrose, 5 g L^−1^ sodium chloride, and 2.5 g L^−1^ disodium phosphate, dissolved in deionized water. Cryogenic stocks were prepared using an actively growing BHI culture after 24 h, by mixing 50 vol% culture broth with 50 vol% oxygen depleted sucrose solution (500 g L^−1^) and freezing 1.8 mL aliquots at −80 °C. A defined minimal medium with glucose (DMM‐G) was used for all cultivations. Defined minimal medium with glucose composition was based on Varel and Bryant^20^ and Lück and Deppenmeier^13^ with 3‐(N‐morpholino)propanesulfonic acid (MOPS) buffer instead of bicarbonate buffer. If not stated otherwise, DMM‐G medium components were obtained from Carl Roth (Karlsruhe, Germany). DMM‐G medium contained 13 individual stock solutions: Base components (pH 7.4), glucose, calcium chloride, magnesium chloride, iron(II) sulfate, SL6‐trace elements, Wolin's vitamin solution,^43^ butyrate, vitamin K1, hemin, L‐cysteine hydrochloride, and MOPS buffer (pH 7.4). Some components were light‐ or temperature‐sensitive, so the stock solutions were stored individually. The base components stock (see Table S1) consisted of ammonium chloride, dipotassium phosphate, monopotassium phosphate, and sodium chloride. The SL6‐trace elements comprised boric acid, cobalt(II)chloride hexahydrate, copper(II)chloride dihydrate, manganese(II)chloride tetrahydrate (Merck, Darmstadt, Germany), nickel(II)chloride, sodium molybdate dihydrate, and zinc sulfate heptahydrate (Merck, Darmstadt, Germany) and were set to pH 7.4 with 5 M sodium hydroxide. The Wolin's vitamin stock solution consisted of α‐lipoic acid, biotin, folate (Sigma Aldrich, St. Louis, USA), nicotinamide, p‐aminobenzoic acid (Sigma Aldrich, St. Louis, USA), pantothenic acid (AppliChem, Darmstadt, Germany), pyridoxine hydrochloride (Sigma Aldrich, St. Louis, USA), riboflavin (Sigma Aldrich, St. Louis, USA), thiamine hydrochloride and vitamin B12. Table S1 lists the final concentrations of all components in the DMM‐G medium. Base components, glucose, calcium chloride, magnesium chloride, iron(II)sulfate, and SL6‐trace elements stocks were sterilized at 121 °C for 20 min. The remaining stock solutions were heat‐sensitive and were, therefore, sterile‐filtered with 0.22 μm polyethersulfone filters (Merck, Darmstadt, Germany). Reducing agent L‐cysteine was sterile‐filtered and stored anaerobically in a serum bottle with a nitrogen atmosphere, to prevent premature oxidation. Wolin's vitamin solution, vitamin K1, and hemin were stored light‐protected at 4 °C after sterilization. All other stock solutions were stored at room temperature. All procedures were carried out as previously described in Keitel et al.^44^

### 2.2 Preparation of oxygen depleted media

To prepare the oxygen depleted pre culture media and oxygen depleted sucrose solution, these were filled into serum bottles and sealed gas‐tight with a rubber stopper and clamp. Afterwards the serum bottles were gassed with N_2_ (99,999% purity) for 20 min, to ensure oxygen depleted conditions. To prepare oxygen depleted main culture medium, the different DMM‐G mixtures were prepared aerobically under a clean bench. Afterwards, the media mixtures were inserted into the anaerobic bench. The anaerobic bench had a gas atmosphere of 2% H_2_, 7% CO_2_, and 91% N_2_. The media mixtures were then distributed in 48 round well microtiter plates (MTP) with a transparent bottom (MTP‐R48‐B, Beckman Coulter GmbH, Baesweiler, Germany). All wells were filled with a volume of 1.9 mL medium each. Next, 4 μL L‐cysteine as a reducing agent was added to each well. After 15 min equilibration time with the anaerobic atmosphere, oxygen depleted conditions were reached.

### 2.3 Preculture

Precultures were carried out in 250 mL serum bottles filled with 50 mL oxygen depleted DMM‐G medium. The serum bottles were gassed with N_2_ (99.999% purity) for 20 min, to ensure an anaerobic atmosphere. Next, CO_2_ (99.999% purity) was added to the gas tight sealed serum bottles with a sterile syringe, to generate a headspace concentration of 10 vol% CO_2_. Afterwards, 0.1 mL L‐cysteine was added as a reducing agent. In the final step, 500 μL cryogenic pure culture of *P. vulgatus* was added to the serum bottles. The serum bottles were cultivated at 37 °C with a shaking frequency of 100 rpm and a shaking diameter of 50 mm for 24 h.

### 2.4 Main culture

**FIGURE 1:**
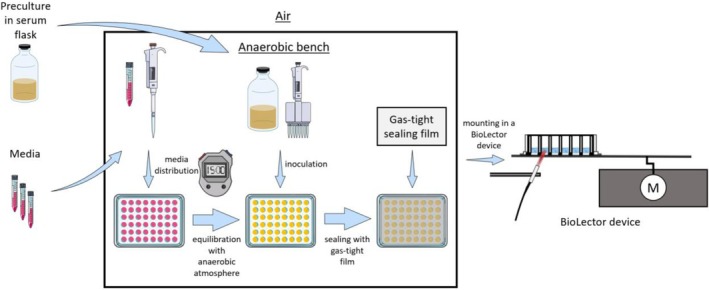
Schematic illustration of experimental procedure for anaerobic microtiter plate cultivations, from serum bottle to BioLector device. For each medium variation, a separate tube was prepared, before inserting it into the anaerobic bench. The gas atmosphere within the anaerobic bench consisted of 2% H_2_, 7% CO_2_, and 91% N_2_. The gas tightness of the microtiter plate was ensured throughout the experiment with the chosen procedure, as proven by Figure S1. Parts of the figure were drawn by using pictures from Servier Medical Art. Servier Medical Art by Servier is licensed under a Creative Commons Attribution 3.0 Unported License. (https://creativecommons.org/licenses/by/3.0/).

The main culture was prepared according to Figure 1 on an anaerobic bench. Each well of the MTP containing 1.9 mL oxygen depleted media was inoculated with 100 μL preculture. Samples for initial offline analysis were prepared simultaneously. The MTP was then sealed with a gas‐tight film (EN77.1, Excel Scientific, Victorville, USA) and additional duct tape (Tesa SE, Norderstedt, Germany), to fix the gas‐tight film. Afterward, the MTP was removed from the anaerobic bench. This setup was gas‐tight and anaerobic, as proven in Figure S1 with a pilot test with dissolved oxygen tension (DOT) optodes.

The MTP was mounted into an in‐house built BioLector for monitoring fluorescence and scattered light with a Fluoromax‐4 spectrometer (HORIBA Jobin‐Yvon GmbH, Bernsheim, Germany, see Figure 1) in MTPs, as described in detail by Wandrey et al.^45^ and Samorski et al.^46^ LabVIEW software developed by ZUMOLab GmbH (Wessling, Germany) was used to control the device and obtain the data. Quasi‐continuous and non‐invasive measurement of scattered light and fluorescence in 48‐well MTPs is enabled with the BioLector device. The MTP was shaken at 600 rpm with a shaking diameter of 3 mm at 37 °C. Scattered light measurement was carried out with an excitation wavelength (ex) of 650 nm and an emission wavelength (em) of 650 nm. NADH fluorescence was measured at ex/em: 340/460 nm and riboflavin fluorescence at ex/em: 450/525 nm. NADH and riboflavin are both intracellular fluorescence proteins, which can easily be measured in a BioLector device. Their evaluation have been conducted in many investigations of microbial cultivations.^46^, ^47^, ^48^ In this work, these signals were measured, to have another marker signal in addition to scattered light intensity, correlating with biomass concentration. NADH and riboflavin fluorescence are independent of the scattered light signal and, thus, unaffected by changes in the medium turbidity or morphology of the bacterium.^46^ NADH acts as the main hydrogen donor for many enzymes^46^ and can be regarded as an indicator for the redox conditions of the microorganism.^46^, ^47^, ^48^, ^49^, ^50^, ^51^, ^52^ Riboflavin is an important vitamin and part of cofactors of several enzymes.^53^ An integration time of 1000 ms was used for scattered light intensity and 600 ms for NADH and riboflavin fluorescence with a slits width (ex and em) of 4 nm for all three parameters.

### 2.5 Data adjusting

The intensity of all online data was background adjusted by using the data of the initial measurement cycle of the reference cultivation.

### 2.6 Offline analysis

Samples were taken from individual wells and the optical density (OD_600nm_) was measured at a wavelength of 600 nm using a Genesys20 photometer (Thermo Scientific, Schwerte, Germany). To correlate the optical density and cell dry weight (CDW), the equation $\mathrm{CDW}=0.563∙{\mathrm{OD}}_{600\mathrm{nm}}$, derived in Keitel and Miebach et al. (2023) for *P. vulgatus*, was used. The remaining samples were centrifuged at 14,000 rpm for 5 min, and the supernatant was used for HPLC and pH measurement. The pH was measured with a pH electrode (Mettler‐Toledo, Columbus, USA). The remaining supernatant was stored at −80 °C. For HPLC analysis, samples were thawed and filtered with 0.2 μm cellulose acetate filters (Merck, Darmstadt, Germany). The SCFAs acetate, succinate, lactate, propionate, formate, and glucose were measured by HPLC. An organic acid resin column of 300 × 8 mm dimensions (CS‐Chromatography, Langerwehe, Germany) was used in the HPLC device (Dionex, Sunnyvale, USA), and the column temperature was set to 60 °C. As an eluent, 5 mM H_2_SO_4_ at a flow rate of 0.8 mL min^−1^ was used. UV/VIS and a refractive index detector were used as detectors during HPLC measurement.

### 2.7 Carbon balances

Carbon balances were calculated for all experiments as described in Keitel et al.^44^ with the following Equation 1:

(1) $${\text{Carbon}}_{\text{in}\;X}\left[\frac{\text{mmol}}{L}\right]=\frac{{\text{Carbon molecules}}_{\text{in}\;X}\left[-\right]}{M_{X}\left[\frac{g}{\mathrm{mol}}\right]}∙c_{X}\left[\frac{g}{L}\right]∙1000$$

where *X* is the specific compound, *c*_*x*_ is the concentration [g L^−1^], *M*_*X*_ is the molar mass of the specific compound [g mol^−1^], *Carbon molecules*_*in X*_ is the number of carbon molecules in the specific compound [−], and *Carbon*_*in X*_ is the volumetric molar carbon in the specific compound [mmol L^−1^].

The compounds glucose, acetate, lactate, succinate, propionate, formate, and biomass of every sample were considered. CO_2_ was not measured in the experiments and, therefore, could not be taken into account. Initial and final concentrations of glucose, acetate, lactate, succinate, propionate, and formate were measured by HPLC. The microbial biomass of *P. vulgatus* cells was based on data from Franke and Deppenmeier^17^ of *P. copri* microbial biomass with a content of 48.48% carbon. First, the volumetric molar carbon [mmol L^−1^] for each compound was calculated, and then the values were combined to obtain the total volumetric molar carbon content for every sample. Finally, to obtain relative values for the compounds, the molar carbon value was divided by the total carbon of the sample, as shown in Equation 2:

(2) $${\text{Carbon}}_{\text{Sample}\;n}\;\left[\%\right]=\frac{{\text{Carbon}}_{\text{in}\;X,S\text{ample}\;n}\left[\frac{\text{mmol}}{L}\right]}{{\text{Total Carbon}}_{\text{Sample}\;n}\;\left[\frac{\text{mmol}}{L}\right]}$$

where *sample n* is designated to a specific sample number in a specific experiment, *Carbon*_*in X, sample n*_ is the volumetric molar carbon of the specific compound in sample n [mmol L^−1^], and *Total Carbon*_*sample n*_ is the sum of all carbon in this sample n [mmol L^−1^], as previously described in Keitel et al.^44^

### 2.8 Software

All graphs were created with OriginPro® version 2021 from OriginLab Corporation (Massachusetts, USA).

## 3 RESULTS AND DISCUSSION

### 3.1 Reference cultivation

In the first experiment, a reference cultivation of *P. vulgatus* was performed, to observe its growth and organic acid production.

**FIGURE 2:**
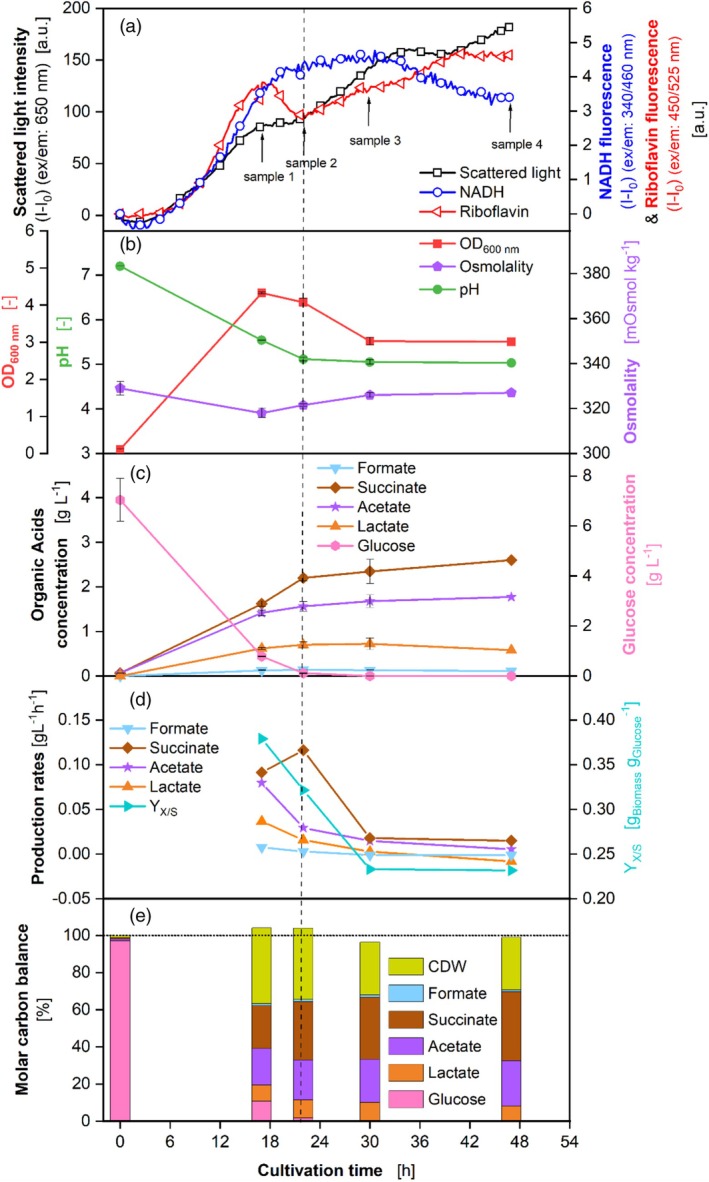
Cultivation of *P. vulgatus* with online data and data from offline sampling in a BioLector device (a) Scattered light, riboflavin & NADH fluorescence intensity. For clarity, only every 10^th^ measuring point is shown as a symbol. For clarity, only one curve is shown for each scattered light, riboflavin & NADH fluorescence. All seven replicates can be found in Figure S2. Offline data were obtained from the same MTP used for online measurement. Wells, which were sampled, were not further used. Error bars indicate maximum and minimum values of biological duplicates. After 47 h, only one sample was taken. (b) OD_600 nm_, pH, and osmolality in duplicates. (c) Produced organic acids and glucose in duplicates. Propionate could not be detected during HPLC measurement. (d) Production rates of the organic acids and yield (Y_X/S_) of g_Biomass_ per g_Glucose_. (e) Carbon balance in % over the course of the cultivation. The start of the fermentation was set to 100%. Horizontal dotted line in (e) highlights 100% of the molar carbon balance. Vertical dashed line is added for clarity, to highlight the time, when glucose is exhausted. Microscopic images of samples can be found in Figure S3. 48‐round‐well microtiter plate, Medium = DMMG, c_Glucose_ = 8 g L^−**1**^, c_buffer_ = 100 mM MOPS, T = 37 °C, *n* = 600 rpm, V_L_ = 2 mL, gas mix = 2% H_2_, 7% CO_2_ and 91% N_2_.

Figure 2a displays the scattered light intensity and NADH and riboflavin fluorescence. For clarity, only one curve for each of the online monitoring signals of scattered light intensity, NADH, and riboflavin fluorescence is shown in Figure 2a. In Figure S2, all replicates are displayed. The scattered light (Figure 2a) shows plateaus between 17–22 h and 34–39 h. The second increase after the first plateau starts after glucose is completely consumed (Figure 2c, vertical dashed line). The NADH curve (Figure 2a) does not display these plateaus but increases strongly until a cultivation time of 20 h. After 29 h, the curve decreases slightly. The decline may be caused by morphological changes or autolysis, as described by Kunze et al.^54^ for aerobic microorganisms.

The riboflavin curve (Figure 2a) rises until 17 h. Subsequently, it decreases until 22 h and increases until the end of the cultivation. The riboflavin signal seems to be very sensitive to extracellular influences (Figure S2c). Its behavior cannot be explained by the position of the used well on the microtiter plate, for example, caused by leakages. The decrease in the riboflavin curve occurs shortly before the glucose is completely consumed. As the OD_600nm_ (Figure 2b) also decreases at that point, autolysis may be an explanation. Another reason for the plateaus in the scattered light and the decrease in riboflavin signal are morphological changes. Morphological changes are presented in Figure S3 by microscopic images. Eley et al.^55^ already described morphological changes in *P. vulgatus*. Low pH values could be a reason for the morphological changes. Figure 2b reveals the optical density (OD_600nm_), pH values, and osmolality. The pH value declines substantially from an initial value of 7.2 to a final value of 5.0 at 22 h, corresponding to a linear SCFA formation (Figure 2c), and remains constant afterward. At 17 h, the pH value (Figure 2b) attains inhibiting levels for *P. vulgatus* with a pH value of 5.5. The literature demonstrated an inhibitory effect of pH values below 6.0 on *P. vulgatus* and that growth stops entirely at a pH value below 5.3.^8^, ^56^ The OD_600nm_ rises until 17 h and decreases until 30 h. Afterward, the OD_600nm_ remains constant. The osmolality remains around the same value throughout the cultivation, between 318 and 329 mOsmol kg^−1^. The osmolality of the medium (~318 mOsmol kg^−1^) is quite low compared to other media. While LB‐medium has a lower osmolality (240 mOsmol kg^−1^), most media, for example, for the aerobic bacterium *Corynebacterium glutamicum* (540 mOsmol kg^−1^) or Syn6‐MES medium (660 mOsmol kg^−1^), for example, for yeast cultivation, have significantly higher osmolalities.^57^

The organic acids and glucose are shown in Figure 2c. At the end of the cultivation 2.6 g L^−1^ (22.02 mM L^−1^) succinate, 1.77 g L^−1^ (30.03 mM L^−1^) acetate, 0.58 g L^−1^ (6.54 mM L^−1^) lactate, and 0.11 g L^−1^ (2.43 mM L^−1^) formate were formed. HPLC measurement could detect no propionate. Formate production is low, while succinate, acetate, and lactate concentrations rise throughout the cultivation, with succinate being the acid produced in the highest amounts. Most acids are produced until 22 h. Succinate even increases after glucose is already depleted. During this time, lactate decreases slightly. Lactate might be consumed by *P. vulgatus* to produce more succinate. Succinate production is a mechanism for *P. vulgatus* to store CO_2_, so it can be released when CO_2_ is scarce.^7^ An inhibiting factor adding to the inhibiting pH value for *P. vulgatus* might be a product inhibition by the organic acids formed, as from 8 g L^−1^ glucose, in total 5.1 g L^−1^ organic acids are formed. Glucose decreases until it is completely consumed at around 22 h. However, the decline is mitigated between ~17 and 22 h, possibly due to a pH inhibition.

The molar carbon balance (calculated according to equations 1 and 2) in Figure 2d is closed, with a maximum deviation of 4.0%, demonstrating that all significant products contributing carbon to the balance have been evaluated. However, CO_2_ could not be measured in the BioLector device and is, therefore, not part of the carbon balance. These results suggest that CO_2_ is only formed in quite limited quantities, which was already shown by Keitel et al.^44^ However, part of the gap in the balance can be explained by the CO_2_ formed by the microorganism, which is not measured and quantified in this work. The biomass accounts for 28.4% of the total carbon at the end of the experiment.

### 3.2 Influence of different glucose concentrations

To determine the influence of different media components, first, the glucose concentration was varied in the following experiments.

**FIGURE 3:**
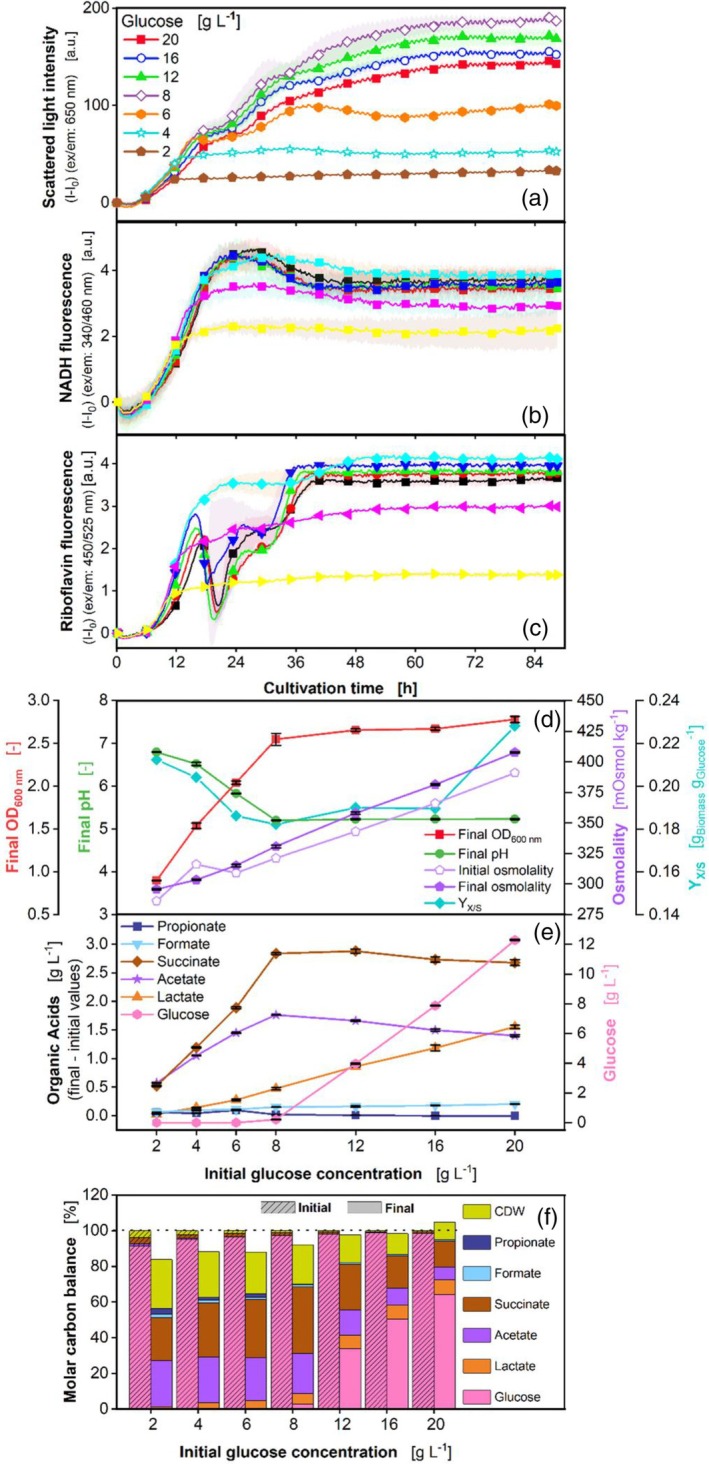
Effect of different glucose concentrations on *P. vulgatus* in a BioLector device. Online data of (a) Scattered light, (b) NADH, and (c) Riboflavin fluorescence intensity. For clarity, only every 10^th^ measuring point is shown as a symbol. Shadows indicate standard deviation of four biological replicates. Due to high measurement accuracy, the shadows are partially barely visible. Offline data of (d) Final OD_600 nm_, final pH, initial and final osmolality, and yield (Y_X/S_) of g_biomass_ per g_glucose_ consumed; (e) Produced organic acids including propionate, formate, succinate, acetate, lactate, and glucose; (f) Carbon balance in % over the applied glucose concentrations. Lines and symbols represent average values of four biological replicates. The start of the fermentation was set to 100%. Horizontal dotted line highlights 100% of the molar carbon balance. 48‐round‐well microtiter plate, medium = DMMG, c_buffer_ = 100 mM MOPS, T = 37 °C, n = 600 rpm, V_L_ = 2 mL, initial OD_600 nm_ = 0.13, initial pH after inoculation = 7.17–7.36, gas mix = 2% H_2_, 7% CO_2_ and 91% N_2_.

The scattered light intensity, disclosed in Figure 3a, shows increasing final values of the curves for glucose concentrations of 2 to 8 g L^−1^. Between 12 and 20 g L^−1^, the endpoints of the curves decrease. One reason for this behavior may be the higher osmolality (Figure 3d), which may lead to a prolonged lag phase and lower viability in aerobic microorganisms.^58^, ^59^ In this experiment, the higher osmolalities might lead to a lower growth rate. Mille et al.^59^ explain several mechanisms that affect the cell during osmotic stress: (1) toxicity of the concentration of dissolved particles in the cell, (2) water efflux across the membrane combined with (3) volume variations of the cells due to the osmotic mass transfer.

After 12 h of cultivation, the curves of glucose concentrations higher than 4 g L^−1^ fan out. The curves of glucose concentrations higher than or equal to 8 g L^−1^ show the same behavior, by forming two plateaus, as observed in the reference cultivation (Figure 2a). The occurrence of these plateaus at higher glucose concentrations might be caused by morphological changes, due to the lowered pH or because of increased acid concentrations. In the NADH fluorescence signal (Figure 3b), there is no evidence for these plateaus at any glucose concentration. This strengthens the speculation that morphological changes occur. The riboflavin fluorescence (Figure 3c) curves of glucose concentrations higher than 6 g L^−1^ show the same decline and progression as in the reference cultivation (Figure 2), with a high standard deviation. This behavior may indicate that the riboflavin production of *P. vulgatus* is sensitive to lowered pH or high levels of organic acids. The fluorescence quantum yield of riboflavin should not have been influenced by the low pH value, as it remains maximal in a pH range of 4.3 to 9.3, according to Drössler et al.^60^

Figure 3d depicts the final OD_600nm_, the final pH, and the initial and final osmolality. While the final OD_600nm_ increases in the range of 2 to 8 g L^−1^ glucose, the final pH value decreases within that range. Between 8 and 20 g L^−1^ glucose, final OD_600nm_, and final pH each remain around the same level, while the final scattered light value (Figure 3a) decreases between 8 and 20 g L^−1^. According to scattered light intensity and glucose (Figure 3e), 8 g L^−1^ glucose can be consumed. As the final pH values of 8 to 20 g L^−1^ all attain the exact value of 5.2, the low pH value is the most likely reason why growth stops after consuming 8 g L^−1^ glucose.

The initial and final osmolalities rise with increasing glucose concentrations. Considering the organic acids in Figure 3e, propionate, and formate production are low over all glucose concentrations. Lactate rises with increasing glucose concentration, while acetate and succinate only increase until an initial glucose concentration of 8 g L^−1^ is reached. Lactate production may be the easiest way for *P. vulgatus* to balance the production of redox equivalents,^36^ as it is the most straightforward metabolic pathway, requiring only a lactate dehydrogenase.^13^ Balancing the production of redox equivalents might be especially valuable, considering the increasing total acid concentration. The acid concentration increases from an initial glucose concentration of 8 with 5.2 g L^−1^ total acids to an initial glucose concentration of 20 g L^−1^ glucose with a total acid concentration of 5.8 g L^−1^, respectively. The low final pH in combination with the high amount of produced acids stresses the cells. Succinate is the most produced acid at 4 g L^−1^ or higher glucose concentrations. The highest succinate yield of 0.56 mol succinate/mol using glucose was reached with an initial glucose concentration of 8 g L^−1^. Compared to other succinate producers, such as *A. succinogenes* (1.42 mol succinate/mol glucose),^61^ *P. vulgatus* is still underperforming. Yet, P. vulgatus is not able to outperform current industrial lactic or acetic acid producers, which achieve titers of 93.09 g L^−1^ acetic acid at a formation rate of 1.83 g L^−1^ ‐h^−1^,^62^ respectively, 34.19 g L^−1^ lactic acid at a formation rate of 4.57 g L^−1^ h^−1^.^63^ However, it gains relatively high yields for a non‐GMO. The molar carbon balance (Figure 3f) is closed with a maximum deviation of 16.2%. The molar carbon balance has a higher deviation with lower glucose concentrations. This behavior might be due to higher errors in HPLC measurement, caused by lower concentrations of the compounds. The CDW accounts for between 27% (2 g L^−1^ glucose) and 10% (20 g L^−1^ glucose) of the total carbon. CO_2_ is not measured and does not play a significant role in the carbon balance of *P. vulgatus*.^44^

### 3.3 Influence of different initial osmolalities

In the next step, the influence of different initial osmolalities on the cultivation of *P. vulgatus* was investigated.

**FIGURE 4:**
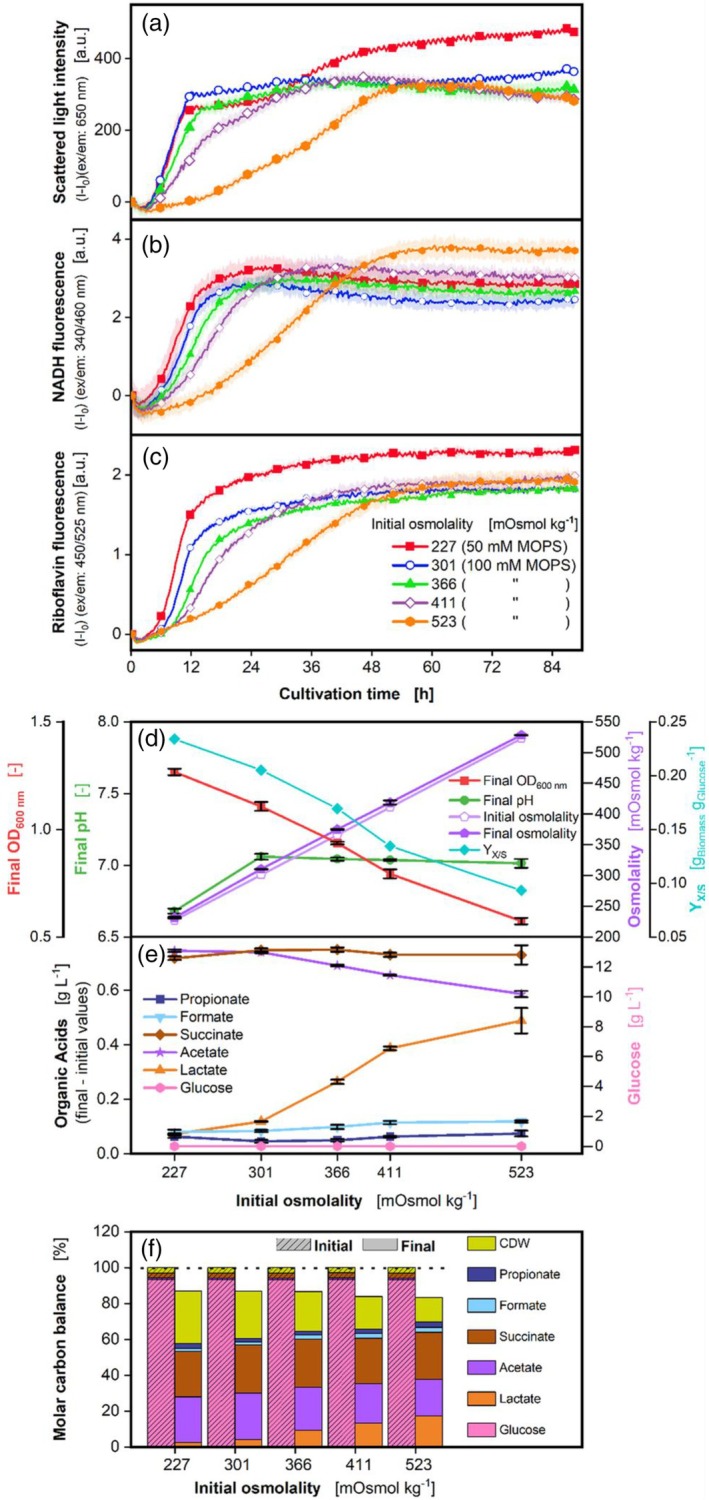
Effect of different initial osmolalities adjusted by NaCl on *P. vulgatus* in a BioLector device. Online data of (a) Scattered light, (b) NADH, and (c) Riboflavin fluorescence intensity. For clarity, only every 24^th^ measuring point is shown as a symbol. Shadows indicate standard deviations of four biological replicates. Due to high measurement accuracy, the shadows are partially barely visible. Offline data of (d) Final OD_600 nm_, final pH, and initial, final osmolality, and yield (Y_X/S_) of g_biomass_ per g_glucose_ consumed; (e) Produced organic acids including propionate, formate, succinate, acetate, lactate, and glucose; (f) Carbon balance in % over the osmolality of the medium. Lines and symbols represent average values of four replicates. The start of the fermentation was set to 100%. Horizontal dotted line highlights 100% of the molar carbon balance. 48‐round‐well microtiter plate, medium = DMMG, c_Glucose_ = 2.7 g L^−1^, c_buffer_ = 100 mM MOPS, except 227 mOsmol kg^−1^, where 50 mM MOPS was needed to reach lower osmolality, NaCl was added to 366, 411 and 523 mOsmol kg^−1^, to adjust osmolality, T = 37 °C, n = 600 rpm, V_L_ = 2 mL, initial OD_600 nm_ = 0.13, initial pH after inoculation = 7.2–7.3, gas mix = 2% H_2_, 7% CO_2_, and 91% N_2_.

The initial osmolality was changed by adding NaCl in different concentrations to the medium. The scattered light intensity, displayed in Figure 4a, reveals a shallower slope of the curves, the higher the initial osmolality in the range 301–523 mOsmol kg^−1^. A total of 227 and 301 mOsmol kg^−1^ show the same slope. The highest final scattered light intensity is attained with the lowest osmolality (227 mOsmol kg^−1^). *P. vulgatus* seems to be quite sensitive against osmotic pressure, concerning its growth rate, as already observed in the experiment with different glucose concentrations (Figure 3). The same slope profile as in the scattered light can be seen in the curves of the NADH and riboflavin fluorescence (Figure 4b,c). Changes in osmolality often occur in the intestine and can be caused by disease, diet, or alcohol consumption.^1^ In addition, the microbiota itself also changes the osmolality in the gut by degrading the mucosal layer or SCFA production. The osmolality of stool is in general about 290 mOsmol kg^−1^.^64^ In a study by Ng et al.^1^ in a liquid culture experiment in 96‐well plates in an anaerobic chamber, *P. vulgatus* showed a decreased growth rate at 890 mOsmol kg^−1^, but not at 440 mOsmol kg^−1^. A total of 440 mOsmol kg^−1^ was the lowest osmolality tested for *P. vulgatus* in the study by Ng et al.^1^ In this study, the growth rate is shown to decrease already at 366 mOsmol kg^−1^, but the decrease is particularly pronounced between 411 and 523 mOsmol kg^−1^. Thus, the results of the study of Ng et al.^1^ and this study for *P. vulgatus* agree well. In contrast, in the study of Ng et al.,^1^ other gut bacteria, such as *Lactobacillaceae*, had high growth rates up to osmolalities of 1800 mOsmol kg^−1^. They further observed that in *Bacteroidaceae* and *Bifidobacteriaceae*, heterogeneities were evident among strains in response to high osmolalities.^1^ For example, the *P. vulgatus* related strain *B. thetaitaomicron* still showed high growth rates at osmolalities up to 1176 mOsmol kg^−1^. Figure 4d discloses the final OD_600nm_, final pH, and initial and final osmolality. The final OD_600nm_ is declining with increasing osmolality.

The final pH is lowest for the smallest initial osmolality and remains on the same level between 301 and 523 mOsmol kg^−1^. The reason for the low pH obtained at an osmolality of 227 mOsmol kg^−1^ is the lower MOPS concentration of 50 mM. The final osmolality increases in the same range as the initial osmolality. The data of organic acids (Figure 4e) reveal that low amounts of propionate and formate have been formed at all initial osmolalities. For the lowest osmolality, the most produced acid is acetate. For the higher osmolalities, it is succinate. Lactate formation rises with increasing osmolality. The increasing lactate formation with increasing initial osmolalities is again an easy way for *P. vulgatus* to balance the production of redox equivalents.^13^, ^36^ Lactate production seems to be a sound strategy if *P. vulgatus* is cultivated under stressful conditions. Correspondingly, acetate production decreases with increasing osmolality, while succinate production remains constant. However, the total acid production increases with increasing osmolalities from 1.7 to 2.0 g L^−1^ (Table S2). As biomass production decreases with increasing osmolalities, more carbon is funneled into organic acid production.

The glucose (Figure 4e) is completely consumed for all initial osmolalities. These results can be compared to a similar experiment with *P. vulgatus* by Keitel et al.^44^ with different initial osmolalities carried out in a RAMOS device (shake flask scale with constant gassing with 99 vol% N_2_ and 1 vol% CO_2_). Gas production of *P. vulgatus* was very low during the cultivation in the RAMOS device,^44^ so no high gas production is expected while cultivating *P. vulgatus* in the BioLector device. In the previous study of Keitel et al.,^44^ longer cultivation times could be observed with increasing initial osmolalities. However, with increasing osmolalities, increasing final pH values and OD_600nm_ were visible in the previous study. This behavior contrasts with the results of this study. One possible reason may be the constant gassing in the RAMOS device, compared to the changing gas atmosphere in the gas‐tight atmosphere of the microtiter plate. As mentioned in the introduction, *P. vulgatus* can form hydrogen.^65^ The bacterium can possibly not manage high levels of hydrogen, which have been gassed out in the RAMOS device, but not in the MTPs in this study. Another reason could be the higher MOPS concentration of 100 mM used in this study (except 227 mOsmol kg^−1^, where 50 mM MOPS were used, Figure 4) compared to 50 mM used in the previous study. Wetzstein and Gottschalk (1985)^66^ showed for genetically related *B. amylophilus* that increased NaCl concentrations up to 90 mM increased growth. As in this study, NaCl was used to adjust the osmolality, but here higher MOPS concentrations of 100 mM were used. Therefore, the fraction of NaCl contributing to the same osmolalities was lower than in the previous study. As *P. vulgatus* can profit from increasing NaCl concentrations,^18^, ^67^ the higher NaCl concentrations can improve biomass growth.

The molar carbon balance (Figure 4f) is closed, with a maximum deviation of 16.7%. CDW accounts for 14% (523 mOsmol kg^−1^) to 29% (227 mOsmol kg^−1^) of the total carbon. Concluding this set of experiments, an increased initial osmolality in the shown range harms biomass production but positively affects total acid production, especially lactate formation.

### 3.4 Influence of different carbon sources

The following experiments examined the growth and organic acid production of *P. vulgatus* with different carbon sources.

**FIGURE 5:**
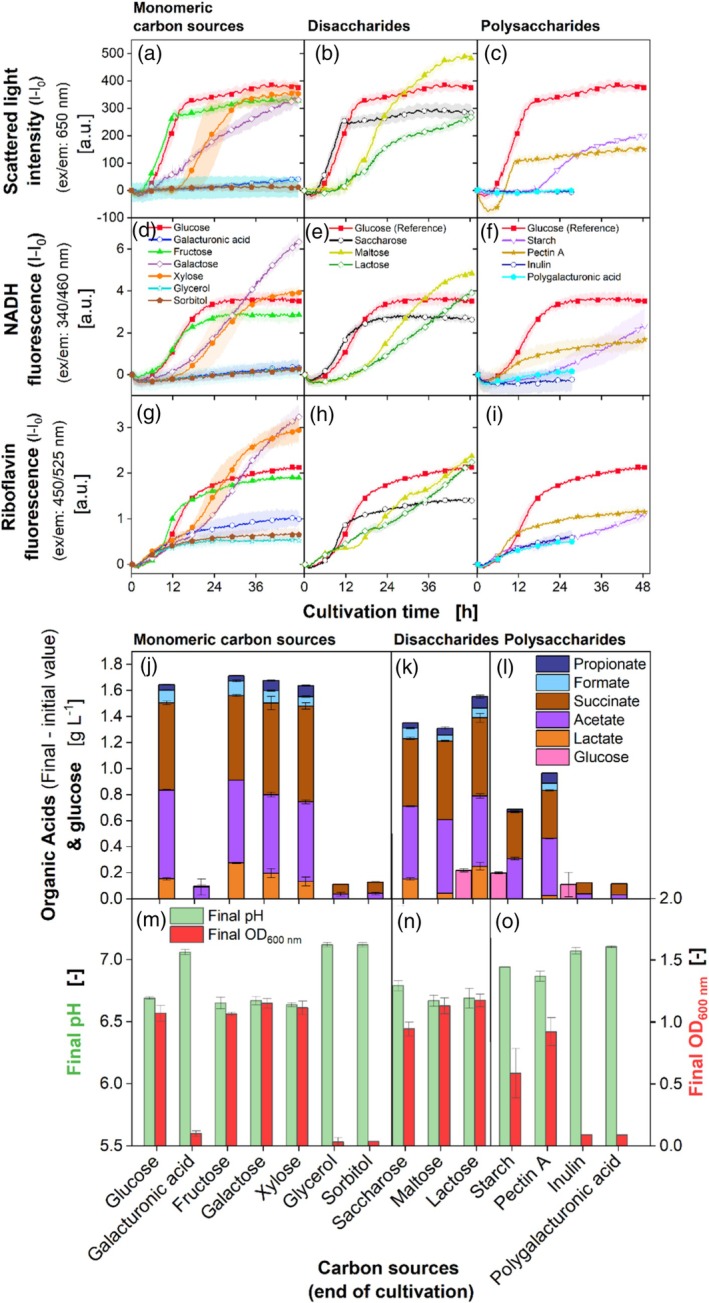
Effect of carbon sources on *P. vulgatus* in a BioLector device. First column: Monomeric carbon sources, second column: Disaccharides, third column: Polysaccharides; Online average data of (a), (b), (c) Scattered light, (d), (e), (f) NADH and (g), (h), (i) Riboflavin fluorescence intensity. For clarity, only every 24^th^ measuring point is shown as a symbol. Shadows indicate standard deviations of four biological replicates. Due to high measurement accuracy, the shadows are partially barely visible. The variability of the lag‐phase of strains grown on xylose and the comparison with glucose are shown in Figure S5. Cultivations with inulin and polygalacturonic acid were terminated earlier because no growth was detected. Preculture was grown on glucose. Offline data of (j), (k), (l) Produced organic acids including propionate, formate, succinate, acetate, lactate, and glucose; (m), (n), (o) Final OD_600 nm_ and final pH. 48‐round‐well microtiter plate, medium = DMMG, c_Glucose_ = 2.7 g L^−1^ and molar carbon equivalents for other carbon sources, c_buffer_ = 100 mM MOPS, T = 37 °C, n = 600 rpm, V_L_ = 2 mL, initial OD_600 nm_ = 0.13, initial pH after inoculation = 7.0–7.1, gas mix = 2% H_2_, 7% CO_2_ and 91% N_2_.

The scattered light and NADH fluorescence during the growth on the monomeric carbon sources (Figure 5a,d) galacturonic acid, glycerol, and sorbitol remain close to zero. Growth on xylose shows a longer lag phase than the other monomeric carbon sources. Xylose is a frequent building unit of lignocellulosic biomass and part of the side chain of pectin.^24^, ^68^ Probably, enzyme induction and expression for xylose utilization must be carried out first.

The scattered light during growth on galactose has a lower slope than during growth on glucose, fructose, and xylose, but reaches the same final value and OD_600nm_ (Figure 5m). Galactose is a common carbon source in the intestine since it is a building block of plant cell walls, mucin, and part of the side chain of pectin.^24^, ^32^, ^69^, ^70^ The reason for the slower growth is the rather complex galactose degradation pathway in *P. vulgatus*, discovered by Hobbs et al.^32^ The NADH fluorescence during growth on the monomeric carbon sources (Figure 5d) reveals the same trends as the scattered light. The riboflavin fluorescence (Figure 5g) is increasing for growth on all monomeric carbon sources.

The scattered light intensity of the disaccharides (Figure 5b) discloses that growth on lactose has a lower slope than maltose, saccharose, and glucose, and a longer lag phase, compared to saccharose. Growth on maltose and lactose both reach high OD_600nm_ (Figure 5n). The NADH fluorescence during growth on the disaccharides (Figure 5e) shows the same trends as the scattered light signal. Riboflavin fluorescence (Figure 5h) shows that during growth on saccharose, it increases and reaches a plateau, while during growth on lactose and especially maltose, it exhibits two to three plateaus during the cultivation until it reaches its final level.

Growth on fructose and saccharose displays in all online data a shorter lag phase, with a steep slope, but a lower endpoint than glucose. Most of the complex polysaccharides reaching the large intestine contain fructose as a building unit.^28^ Therefore, fast growth on fructose is not surprising, further confirming the feasibility of fructose as a carbon source for *P. vulgatus*. Saccharose is a product of the sugar cane and sugar beet industry^71^ and could be interesting in serving as a substrate for the sustainable production of organic acids with *P. vulgatus*.

In contrast, the growth on the disaccharide lactose is slow (Figure 5b), which is not surprising, considering that lactose is composed of glucose and galactose, and the growth on galactose (Figure 4a) was slow, too. McCarthy et al.^29^ have shown that α‐glucosidase activities were high when *P. vulgatus* was cultivated on maltose. Apparently, the enzyme is needed to degrade maltose in *P. vulgatus*. The growth on maltose is also slow. Considering that it consists of two glucose units, the bond is the limiting factor here. The cultures on galactose, maltose, and lactose were terminated too early. It is possible that higher concentrations of organic acids would have been reached if the experiment had been performed until the stationary growth phase.

The scattered light intensity and NADH fluorescence during growth on the polysaccharides (Figure 5c,f) show values close to zero for inulin and polygalacturonic acid. Considering the scattered light and NADH data (Figure 5a–f), as well as the OD_600nm_ (Figure 5m–o), no growth of *P. vulgatus* is possible on galacturonic acid, glycerol, sorbitol, inulin, and polygalacturonic acid. The scattered light curve for growth on pectin A shows a longer lag phase than glucose, but increases with the same slope.

The scattered light curve during growth on starch rises after 17 h with a lower slope but higher endpoint value than for growth on pectin A concerning. The riboflavin fluorescence during growth on the polysaccharides (Figure 5i) discloses the same increase for starch, inulin, and polygalacturonic acid. Interestingly, the riboflavin fluorescence during growth on galacturonic acid, polygalacturonic acid, glycerol, sorbitol, and inulin is increasing, despite no biomass growth being observed (as indicated by scattered light intensity and NADH fluorescence). As riboflavin is an essential electron carrier in redox reactions,^72^ it might be proof of the SCFA production that is pursued to a low extent (Figure 5j–l) for the carbon sources, where no growth is possible.

Growth on pectin A has a higher slope and attains the same final riboflavin value as starch. Growth on starch discloses a long lag phase (Figure 5c) and slow growth with a low final OD_600nm_ (Figure 5o). Starch is degraded by amylases to maltose,^73^ followed by degradation to glucose. However, the slow growth on starch indicates a limitation in glucose delivery to the metabolism. An indication of this behavior is the detection of glucose at the end of the experiment with starch (Figure 5l), leading to the assumption that glucose was released when *P. vulgatus* already entered the phase of declining growth. Growth on pectin A was not as effective as on glucose. Pectin A consists of α‐1,4‐linked D‐galacturonic acid with rhamnogalacturonan I and II, containing rhamnose, fucose, xylose, galactose, and other sugars.^24^ As no growth and almost no organic acid production could be detected for pectin's main building block, polygalacturonic acid, and its monomer, galacturonic acid, *P. vulgatus* was probably not able to completely degrade pectin A and used the monomeric sugars like rhamnose, fucose, xylose or galactose as carbon sources. Different studies^24^, ^30^ have shown that *Bacteroides* species and specifically *P. vulgatus* possess enzymes for galacturonic acid degradation, but the induction of the responsible enzymes might take more time or a change in conditions. However, the final OD_600nm_ reached by metabolizing pectin A (Figure 5o) is quite high, probably caused by the turbid nature of the pectin A solution.

Concerning the produced organic acids during growth on monomeric carbon sources (Figure 5j), disaccharides (Figure 5k), and polysaccharides (Figure 5l), the highest total acid productions are obtained during growth on glucose, fructose, galactose or xylose (see Table S2). Considering the succinate yield, production is highest on maltose and lactose with 0.68 mol succinate/mol carbon source. If the cultivation times are extended, higher yields could be achieved with some carbon sources. During this set of experiments, final pH values (Figure 5m–o) stayed above 6.6. Therefore, inhibition by the pH value and, thus, its influence on the results can be excluded.

### 3.5 Influence of different nitrogen sources

In addition to the carbon source, the nitrogen source is important for intestinal bacteria. Thus, different nitrogen sources were tested. To avoid nitrogen limitations, the minimum required nitrogen concentration was determined beforehand (Figure S4).

**FIGURE 6:**
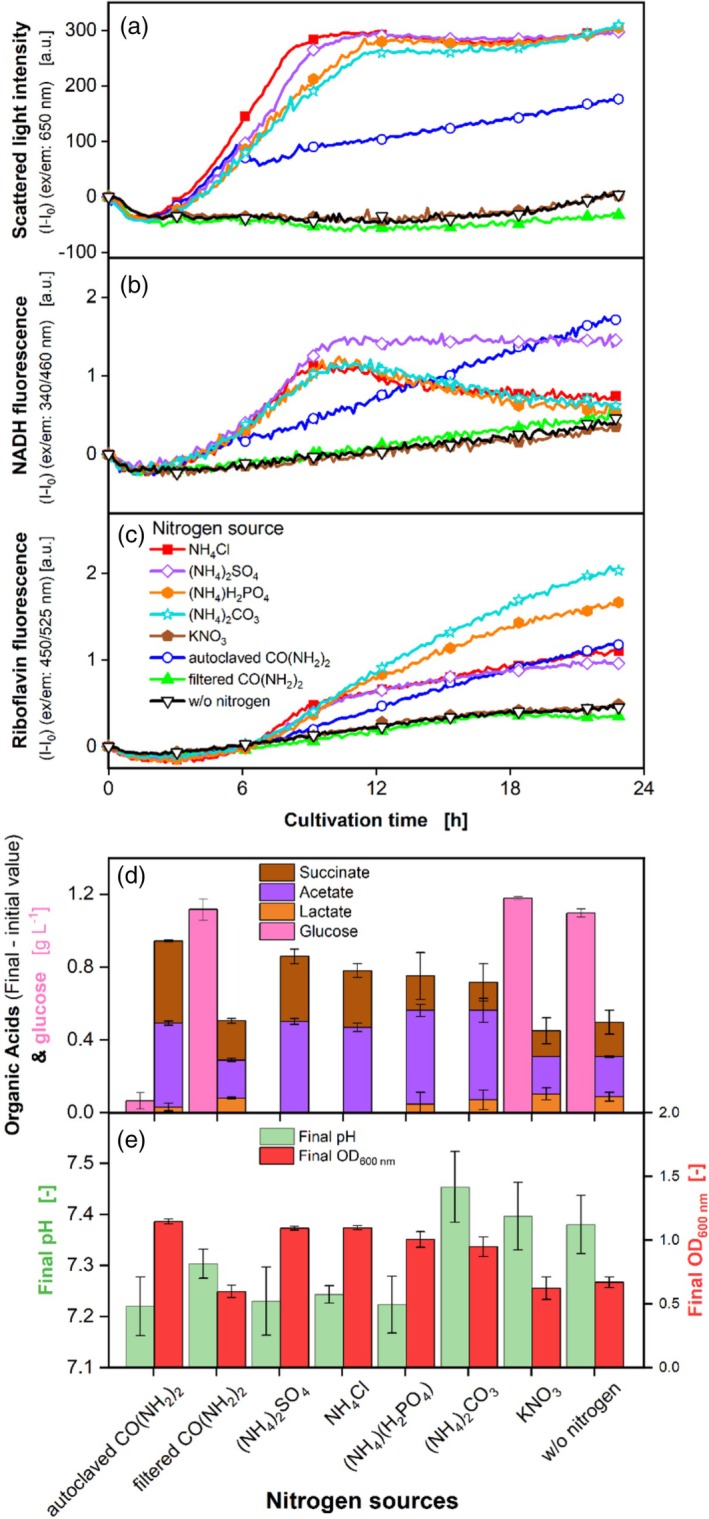
Effect of different nitrogen sources on *P. vulgatus* in a BioLector device. Online average data of three biological replicates of (a) Scattered light, (b) NADH, and (c) Riboflavin fluorescence intensity. Shadows for standard deviations of three biological replicates are not shown for clarity and can be found in Figure S6. For clarity, only every 24^th^ measuring point is shown as a symbol. Offline data of (d) Produced organic acids including succinate, acetate, lactate, and glucose. Propionate and formate could not be detected during HPLC measurement. (e) Final OD_600 nm_ and final pH. 48‐round‐well microtiter plate, medium = DMMG, c_Glucose_ = 2.7 g L^−1^, c_buffer_ = 100 mM MOPS, 0.014 mol L^−1^ N_2_, T = 37°C, n = 600 rpm, V_L_ = 2 mL, initial OD_600 nm_ = 0.13, initial pH after inoculation = 7.11–7.23, gas mix = 2% H_2_, 7% CO_2_ and 91% N_2_.

The scattered light (Figure 6a), NADH (Figure 6b), and riboflavin (Figure 6c) curves remain close to zero for the conditions without nitrogen, with filtered CO(NH_2_)_2_ and KNO_3_. Therefore, only limited growth is possible on these nitrogen sources. The interpretation of the NADH and riboflavin fluorescence signals as indicator for growth on the remaining nitrogen sources is difficult, as the signal errors are quite high (Figure S6). When autoclaved CO(NH_2_)_2_ is used as a nitrogen source, scattered light increases with the same slope as the ammonium compounds, until it turns into a negative trend at 5.7 h. The ammonium compounds have the highest slopes and reach the highest final scattered light. In the NADH fluorescence, the growth on ammonium compounds obtain the highest slopes, but growth on autoclaved CO(NH_2_)_2_ reaches the highest endpoint. Additionally, the highest final OD_600nm_ (Figure 6e) are obtained during growth on autoclaved CO(NH_2_)_2_ and the ammonium compounds. The observed utilization of ammonia sources confirms the observations of Varel and Bryant,^20^ who showed that *P. vulgatus* can metabolize ammonia, but not amino acids. Limited growth is possible on autoclaved CO(NH_2_)_2_, which degrades partially during thermal treatment,^74^, ^75^ making free ammonium accessible for *P. vulgatus*.

Figure 6d unveils the results of HPLC measurement, no propionate or formate was detected. There was no lactate production for the conditions with (NH_4_)_2_SO_4_ and NH_4_Cl and little lactate production for the other nitrogen sources. Acetate is the organic acid produced in the largest amounts during growth on all nitrogen sources, except filtered CO(NH_2_)_2_, where acetate equals succinate formation. The highest total acid production was obtained with growth on autoclaved CO(NH_2_)_2_ (Table S2). Only while growing on the ammonium compounds, the whole glucose was consumed. It is surprising that even with reduced growth and a significant fraction of unmetabolized glucose for cultivation on CO(NH_2_)_2_, KNO_3_, or without nitrogen, the SCFA production was relatively high, compared to the nitrogen sources, where higher growth was obtained (Figure 6d,e). The resting cell phenomenon could be a reason for the higher acid production. It was shown in several studies for other strains.^76^, ^77^ However, a high fraction of lactate was produced during growth on the nitrogen sources with rather low biomass formation. As observed before, lactate seems to be produced, if *P. vulgatus* is cultivated under stressful conditions. Interestingly, the highest fraction of succinate is produced by cultivation with autoclaved CO(NH_2_)_2_, while for the ammonia sources, which provide higher growth, a high fraction of succinate is achieved with (NH_4_)_2_SO_4_ and NH_4_Cl. These two ammonia sources show no production of lactate, which seems to be a sign of less stressful cultivation conditions for *P. vulgatus*. The utilization of the different nitrogen sources lead to significant differences in the final pH value (Figure 6e). It is in the range of 7.2 for autoclaved CO(NH_2_)_2_, filtered CO(NH_2_)_2_, (NH_4_)_2_SO_4_, NH_4_Cl, and (NH_4_)(H_2_PO_4_) and 7.4 for (NH_4_)_2_CO_3_, KNO_3_ and without nitrogen. The differences in growth, however, do not correlate with the pH changes, but are solely dependent on the nitrogen source. Therefore, a pH inhibition can be excluded.

## 4 CONCLUSIONS

This study shows the successful growth and online monitoring of *P. vulgatus* in a shaken bioreactor on MTP scale. In future, this approach can also be transferred to other anaerobic strains, to accelerate screening and characterization of strains tasks for the production of valuable compounds. Furthermore, this kind of cultivation can be used to optimize the organic acid production by *P. vulgatus*. Using glucose as a carbon source, the highest succinate concentrations were gained with a glucose concentration of 12 g L^−1^ (2.9 g L^−1^ succinate), while the highest acetate concentrations were gained with a glucose concentration of 8 g L^−1^ (1.8 g L^−1^ acetate). However, the highest yield for succinate was reached with 8 g L^−1^ glucose and for acetate with 2 g L^−1^ glucose. A lower osmolality was, in general, more beneficial for acid production. Growth on a nitrogen concentration for NH_4_Cl of 0.25 g L^−1^ obtained the highest succinate and acetate concentrations. Among the different nitrogen sources, the cultivation on ammonia compounds attained the highest acid production. Considering the carbon sources, most succinate was formed during growth on xylose or galactose as a carbon source, and most acetate with glucose as the carbon source. However, succinate yield was highest during growth on maltose and lactose, and acetate yield with maltose as a carbon source. Growth on maltose and lactose could outperform the other carbon sources with longer cultivation times. The yields of other carbon sources than glucose could also improve if the preculture is already performed on the carbon source used for the main culture. Minimal amounts of propionate and formate have been formed. Genetic modifications, to enhance the succinate yield and shift acid production from acetate and lactate to succinate, is a future measure to increase the potential of *P. vulgatus* as a platform organism for SCFA production. Isar et al.^78^ and Isar et al.^79^ could improve succinate production with pH control at pH 7.0 with genetically related *B. fragilis*. Fermentations with *P. vulgatus* may also achieve higher succinate titers in an optimized medium and with pH control. Succinate and acetate production could also be increased by optimizing growth on alternative carbon sources, such as lactose or maltose. The aforementioned undefined anaerobic mixed culture, based on organic waste streams, is another promising approach to produce bio‐based SCFA, as recently shown by several groups.^38^, ^39^, ^40^, ^41^

Characterizing cultivation parameters for *P. vulgatus* in axenic culture and examining media variations show the potential of *P. vulgatus* as an SCFA producer. However, it cannot yet compete with industrial producers. *P. vulgatus* can consume a variety of carbon sources, making it a suitable candidate for sustainable SCFA production based on renewable feedstock.

## AUTHOR CONTRIBUTIONS

**Laura Keitel:** Conceptualization; data curation; formal analysis; investigation; methodology; visualization; writing – original draft; writing – review and editing. **Benjamin Schick:** Writing – review and editing. **Gino Pohen:** Investigation. **Stanislav Yordanov:** Investigation. **Jochen Büchs:** Conceptualization; resources; supervision; writing – review and editing.

## FUNDING INFORMATION

This study was funded by the German Federal Ministry of Education and Research (BMBF, Grant number: 031B0846B). Projekt DEAL, supervised by the German Rectors' Conference, supported publication under creative commons license CC‐BY.

## CONFLICT OF INTEREST STATEMENT

The authors have no relevant financial or non‐financial interests to disclose.

### PEER REVIEW

The peer review history for this article is available at https://www.webofscience.com/api/gateway/wos/peer‐review/10.1002/btpr.3526.

## Supporting information


**Data S1.** Supporting Information.

## Data Availability

The data that support the findings of this study are available from the corresponding author upon reasonable request.
