# Supplementary material for: Online monitored characterization of Phocaeicola vulgatus for organic acid production using anaerobic microtiter plate cultivations
Source: Biotechnol Prog. 2024 Dec 20;41(2):e3526. doi: 10.1002/btpr.3526 (PMC12000641; doi:10.1002/btpr.3526)
Supplement: Supplementary file 1 — Data S1. Supporting Information. [file BTPR-41-e3526-s001.docx]

**Supplementary Information**

**Online monitored characterization of *Phocaeicola vulgatus* for organic acid production using anaerobic microtiter plate cultivations**

Laura Keitel^1†^, Benjamin Schick^1†^, Gino Pohen^1^, Stanislav Yordanov^1^ and Jochen Büchs^1*^

^1^ RWTH Aachen University, Chair of Biochemical Engineering (AVT.BioVT), Forckenbeckstraße 51, 52074 Aachen, Germany

^†^ Laura Keitel and Benjamin Schick contributed equally to this work.

* Corresponding author: Jochen Büchs, RWTH Aachen University, Biochemical Engineering (AVT.BioVT), Forckenbeckstraße 51, 52074 Aachen, Germany, Tel.: +49 241 80 24633, Fax: +49 241 80 22635, E-mail: [jochen.buechs@avt.rwth-aachen.de](mailto:jochen.buechs@avt.rwth-aachen.de)

Email addresses:

LK: [laura.keitel@avt.rwth-aachen.de](mailto:laura.keitel@avt.rwth-aachen.de)

BS: Benjamin.Schick@avt.rwth-aachen.de

GP: [gino.pohen@rwth-aachen.de](mailto:gino.pohen@rwth-aachen.de)

SY: [stanislav.yordanov@rwth-aachen.de](mailto:stanislav.yordanov@rwth-aachen.de)

JB: [jochen.buechs@avt.rwth-aachen.de](mailto:jochen.buechs@avt.rwth-aachen.de)

ORCIDS:

LK: 0000-0003-2434-9541

BS: 0009-0007-0577-0652

JB: 0000-0002-2012-3476

**Table S1: Concentration of DMM-G medium components used in this work in alphabetical order**

| Name | Final concentration in DMM-G medium |
| --- | --- |
| α-lipoic acid | 0.05 mg L^-1^ |
| Ammonium chloride | 0.75 g L^-1^ |
| Biotin | 0.02 mg L^-1^ |
| Boric acid | 0.6 mg L^-1^ |
| Butyric acid | 0.176 mg L^-1^ |
| Calcium chloride | 0.026 g L^-1^ |
| Cobalt(II)chloride hexahydrate | 0.4 mg L^‑1^ |
| Copper(II)chloride dihydrate | 0.02 mg L^-1^ |
| Dipotassium phosphate | 2.2 g L^-1^ |
| Folate | 0.02 mg L^-1^ |
| Glucose | 6.0 g L^-1^ |
| Hemin | 1.0 mg L^-1^ |
| Iron(II) sulphate | 1.39 mg L^-1^ |
| L-cysteine hydrochloride | 484 mg L^-1^ |
| Magnesium chloride | 0.1 g L^-1^ |
| Manganese(II) chloride tetrahydrate | 0.06 mg L^-1^ |
| Monopotassium phosphate | 1.7 g L^-1^ |
| Nickel(II)chloride hexahydrate | 0.04 mg L^-1^ |
| Nicotinamide | 0.05 mg L^-1^ |
| P-aminobenzoic acid | 0.05 mg L^-1^ |
| Pantothenic acid | 0.05 mg L^-1^ |
| Pyridoxine hydrochloride | 0.1 mg L^-1^ |
| Riboflavin | 0.05 mg L^-1^ |
| Sodium chloride | 0.9 g L^-1^ |
| Sodium molybdate dihydrate | 0.06 mg L^-1^ |
| Thiamine hydrochloride | 0.05 mg L^-1^ |
| Vitamin B12 | 0.001 mg L^-1^ |
| Vitamin K1 | 9.95 ·10^−5^ % v/v |
| Zinc sulfate heptahydrate | 0.2 mg L^-1^ |

| **Experiment** | **Figure** | **Total acid production [g L^-1^]** | | | **Total lactate production [g L^-1^]** | | | **Total acetate production [g L^-1^]** | | | **Total succinate production [g L^-1^]** | | | **Total propionate production [g L^-1^]** | | | **Total formiate production [g L^-1^]** | | |
| --- | --- | --- | --- | --- | --- | --- | --- | --- | --- | --- | --- | --- | --- | --- | --- | --- | --- | --- | --- |
| **Reference cultivation** | **2** |  |  |  |  |  |  |  |  |  |  |  |  |  |  |  |  |  |  |
| **Glucose concentrations [g L^-1^]** | **3** |  |  |  |  |  |  |  |  |  |  |  |  |  |  |  |  |  |  |
| 2 |  | 1.25 | ± | 9.5E-03 | 0.03 | ± | 1.9E-03 | 0.57 | ± | 1.3E-03 | 0.52 | ± | 1.1E-02 | 0.06 | ± | 5.3E-03 | 0.07 | ± | 5.3E-04 |
| 4 |  | 2.53 | ± | 1.5E-02 | 0.14 | ± | 1.1E-03 | 1.05 | ± | 5.9E-03 | 1.19 | ± | 1.1E-02 | 0.04 | ± | 1.8E-03 | 0.10 | ± | 9.8E-03 |
| 6 |  | 3.82 | ± | 2.2E-02 | 0.28 | ± | 1.1E-02 | 1.45 | ± | 7.2E-03 | 1.89 | ± | 1.6E-02 | 0.09 | ± | 1.5E-03 | 0.11 | ± | 3.9E-03 |
| 8 |  | 5.25 | ± | 1.2E-02 | 0.48 | ± | 1.9E-02 | 1.76 | ± | 4.2E-03 | 2.84 | ± | 1.5E-02 | 0.02 | ± | 1.1E-03 | 0.15 | ± | 3.5E-03 |
| 12 |  | 5.57 | ± | 1.1E-02 | 0.43 | ± | 3.6E-03 | 0.83 | ± | 4.7E-03 | 1.44 | ± | 1.6E-02 | 0.01 | ± | 3.7E-04 | 0.08 | ± | 6.4E-03 |
| 16 |  | 5.59 | ± | 7.1E-03 | 0.40 | ± | 1.4E-02 | 0.50 | ± | 5.7E-03 | 0.91 | ± | 1.3E-02 | 0.00 | ± | 4.7E-05 | 0.06 | ± | 2.3E-03 |
| 20 |  | 5.84 | ± | 8.3E-03 | 0.39 | ± | 5.8E-03 | 0.35 | ± | 3.4E-03 | 0.67 | ± | 1.0E-02 | 0.00 | ± | 0.0E+00 | 0.05 | ± | 8.0E-04 |
| **Initial osmolalities [mOsmol kg^-1^]** | **4** |  |  |  |  |  |  |  |  |  |  |  |  |  |  |  |  |  |  |
| 227 |  | 1.68 | ± | 1.2E-02 | 0.07 | ± | 1.8E-03 | 0.75 | ± | 3.8E-03 | 0.72 | ± | 5.4E-03 | 0.06 | ± | 4.0E-03 | 0.08 | ± | 6.6E-03 |
| 301 |  | 1.73 | ± | 1.1E-02 | 0.12 | ± | 1.3E-03 | 0.74 | ± | 2.6E-03 | 0.75 | ± | 5.4E-03 | 0.05 | ± | 4.5E-03 | 0.08 | ± | 3.2E-03 |
| 366 |  | 1.85 | ± | 7.3E-03 | 0.26 | ± | 6.6E-03 | 0.69 | ± | 2.6E-03 | 0.75 | ± | 6.4E-03 | 0.05 | ± | 3.8E-03 | 0.10 | ± | 6.5E-03 |
| 411 |  | 1.95 | ± | 7.0E-03 | 0.39 | ± | 5.3E-03 | 0.66 | ± | 1.8E-03 | 0.73 | ± | 6.1E-03 | 0.06 | ± | 2.8E-03 | 0.11 | ± | 5.2E-03 |
| 523 |  | 2.00 | ± | 5.3E-03 | 0.49 | ± | 3.8E-02 | 0.59 | ± | 9.6E-03 | 0.73 | ± | 2.9E-02 | 0.07 | ± | 8.8E-03 | 0.12 | ± | 3.2E-03 |
| **NH_4_Cl concentrations [g L^-1^]** | **5** |  |  |  |  |  |  |  |  |  |  |  |  |  |  |  |  |  |  |
| 0.1 |  | 1.62 | ± | 7.6E-03 | 0.63 | ± | 2.2E-02 | 0.50 | ± | 1.0E-02 | 0.38 | ± | 3.5E-02 | 0.05 | ± | 5.7E-03 | 0.06 | ± | 3.7E-03 |
| 0.25 |  | 1.76 | ± | 7.3E-03 | 0.59 | ± | 1.1E-02 | 0.57 | ± | 3.9E-03 | 0.46 | ± | 5.7E-03 | 0.04 | ± | 7.5E-03 | 0.10 | ± | 3.1E-03 |
| 0.5 |  | 1.72 | ± | 1.1E-02 | 0.56 | ± | 1.7E-02 | 0.57 | ± | 6.7E-03 | 0.45 | ± | 3.8E-03 | 0.04 | ± | 8.2E-03 | 0.10 | ± | 2.9E-03 |
| 0.75 |  | 1.64 | ± | 1.0E-01 | 0.55 | ± | 3.5E-02 | 0.54 | ± | 2.8E-02 | 0.42 | ± | 2.5E-02 | 0.04 | ± | 7.5E-03 | 0.09 | ± | 6.6E-03 |

**Table S2: Total acid production, total lactate production, total acetate production, total succinate production, total formiate production (final – initial values) [g L^-1^] of all experiments**

| **Experiment** | **Figure** | **Total acid production [g L^-1^]** | | | **Total lactate production [g L^-1^]** | | | **Total acetate production [g L^-1^]** | | | **Total succinate production [g L^-1^]** | | | **Total propionate production [g L^-1^]** | | | **Total formiate production [g L^-1^]** | | |
| --- | --- | --- | --- | --- | --- | --- | --- | --- | --- | --- | --- | --- | --- | --- | --- | --- | --- | --- | --- |
| **Carbon sources** | **6** |  |  |  |  |  |  |  |  |  |  |  |  |  |  |  |  |  |  |
| Glucose |  | 1.64 | ± | 1.5E-03 | 0.15 | ± | 7.5E-03 | 0.68 | ± | 3.9E-03 | 0.67 | ± | 1.2E-02 | 0.04 | ± | 3.0E-03 | 0.10 | ± | 2.2E-03 |
| Galacturonic acid |  | 0.10 | ± | 5.8E-02 | 0.00 | ± | 0.0E+00 | 0.15 | ± | 5.1E-02 | 0.08 | ± | 7.2E-03 | 0.00 | ± | 0.0E+00 | 0.00 | ± | 0.0E+00 |
| Fructose |  | 1.71 | ± | 6.7E-03 | 0.27 | ± | 4.6E-03 | 0.64 | ± | 7.6E-04 | 0.65 | ± | 6.7E-03 | 0.04 | ± | 2.9E-03 | 0.11 | ± | 6.4E-03 |
| Galactose |  | 1.68 | ± | 6.2E-02 | 0.20 | ± | 2.8E-02 | 0.60 | ± | 1.6E-02 | 0.70 | ± | 4.2E-02 | 0.08 | ± | 3.7E-03 | 0.10 | ± | 4.2E-03 |
| Xylose |  | 1.64 | ± | 5.3E-03 | 0.13 | ± | 2.8E-02 | 0.61 | ± | 1.2E-02 | 0.74 | ± | 2.0E-02 | 0.08 | ± | 4.8E-03 | 0.07 | ± | 4.4E-03 |
| Glycerol |  | 0.11 | ± | 1.2E-02 | 0.00 | ± | 0.0E+00 | 0.04 | ± | 1.1E-02 | 0.07 | ± | 9.4E-04 | 0.00 | ± | 0.0E+00 | 0.00 | ± | 0.0E+00 |
| Sorbitol |  | 0.13 | ± | 1.0E-02 | 0.00 | ± | 0.0E+00 | 0.04 | ± | 8.2E-03 | 0.09 | ± | 2.2E-03 | 0.00 | ± | 0.0E+00 | 0.00 | ± | 0.0E+00 |
| Saccharose |  | 1.35 | ± | 3.4E-03 | 0.15 | ± | 7.1E-03 | 0.56 | ± | 3.0E-03 | 0.52 | ± | 7.5E-03 | 0.04 | ± | 9.0E-04 | 0.08 | ± | 3.0E-03 |
| Maltose |  | 1.31 | ± | 5.2E-03 | 0.04 | ± | 1.1E-03 | 0.57 | ± | 1.2E-03 | 0.60 | ± | 4.1E-03 | 0.05 | ± | 6.6E-03 | 0.05 | ± | 3.8E-04 |
| Lactose |  | 1.55 | ± | 1.4E-02 | 0.25 | ± | 2.4E-02 | 0.54 | ± | 1.3E-02 | 0.60 | ± | 2.7E-02 | 0.09 | ± | 1.0E-02 | 0.07 | ± | 3.6E-03 |
| Starch |  | 0.69 | ± | 1.4E-02 | 0.00 | ± | 0.0E+00 | 0.31 | ± | 7.9E-03 | 0.36 | ± | 6.0E-03 | 0.02 | ± | 2.0E-03 | 0.00 | ± | 0.0E+00 |
| Pectin A |  | 0.96 | ± | 3.2E-03 | 0.02 | ± | 1.7E-03 | 0.44 | ± | 2.7E-03 | 0.37 | ± | 3.4E-03 | 0.08 | ± | 1.3E-03 | 0.06 | ± | 6.5E-04 |
| Inulin |  | 0.12 | ± | 1.5E-03 | 0.00 | ± | 0.0E+00 | 0.04 | ± | 1.4E-03 | 0.09 | ± | 1.6E-04 | 0.00 | ± | 0.0E+00 | 0.00 | ± | 0.0E+00 |
| Polygalacturonic acid |  | 0.11 | ± | 5.0E-04 | 0.00 | ± | 0.0E+00 | 0.03 | ± | 2.1E-04 | 0.08 | ± | 3.1E-04 | 0.00 | ± | 0.0E+00 | 0.00 | ± | 0.0E+00 |
| **Nitrogen sources** | **7** |  |  |  |  |  |  |  |  |  |  |  |  |  |  |  |  |  |  |
| autoclaved CO(NH_2_)_2_ |  | 0.94 | ± | 2.5E-02 | 0.03 | ± | 2.2E-02 | 0.46 | ± | 1.1E-02 | 0.45 | ± | 4.1E-03 | 0.00 | ± | 0.0E+00 | 0.00 | ± | 0.0E+00 |
| filtered CO(NH2)2 |  | 0.50 | ± | 2.8E-02 | 0.08 | ± | 5.4E-03 | 0.21 | ± | 9.4E-03 | 0.22 | ± | 1.4E-02 | 0.00 | ± | 0.0E+00 | 0.00 | ± | 0.0E+00 |
| (NH_4_)_2_SO_4_ |  | 0.86 | ± | 5.5E-02 | 0.00 | ± | 0.0E+00 | 0.50 | ± | 1.6E-02 | 0.36 | ± | 4.0E-02 | 0.00 | ± | 0.0E+00 | 0.00 | ± | 0.0E+00 |
| NH_4_Cl |  | 0.78 | ± | 6.1E-02 | 0.00 | ± | 0.0E+00 | 0.47 | ± | 2.3E-02 | 0.31 | ± | 3.8E-02 | 0.00 | ± | 0.0E+00 | 0.00 | ± | 0.0E+00 |
| (NH_4_)(H_2_PO_4_) |  | 0.75 | ± | 2.9E-02 | 0.05 | ± | 6.5E-02 | 0.51 | ± | 3.5E-02 | 0.19 | ± | 1.3E-01 | 0.00 | ± | 0.0E+00 | 0.00 | ± | 0.0E+00 |
| (NH_4_)_2_CO_3_ |  | 0.72 | ± | 1.9E-01 | 0.07 | ± | 5.3E-02 | 0.49 | ± | 6.7E-02 | 0.15 | ± | 1.0E-01 | 0.00 | ± | 0.0E+00 | 0.00 | ± | 0.0E+00 |
| KNO_3_ |  | 0.45 | ± | 1.0E-01 | 0.10 | ± | 3.3E-02 | 0.20 | ± | 5.0E-05 | 0.14 | ± | 7.1E-02 | 0.00 | ± | 0.0E+00 | 0.00 | ± | 0.0E+00 |
| w/o nitrogen |  | 0.50 | ± | 6.9E-02 | 0.09 | ± | 2.5E-02 | 0.22 | ± | 5.6E-03 | 0.19 | ± | 6.5E-02 | 0.00 | ± | 0.0E+00 | 0.00 | ± | 0.0E+00 |

**
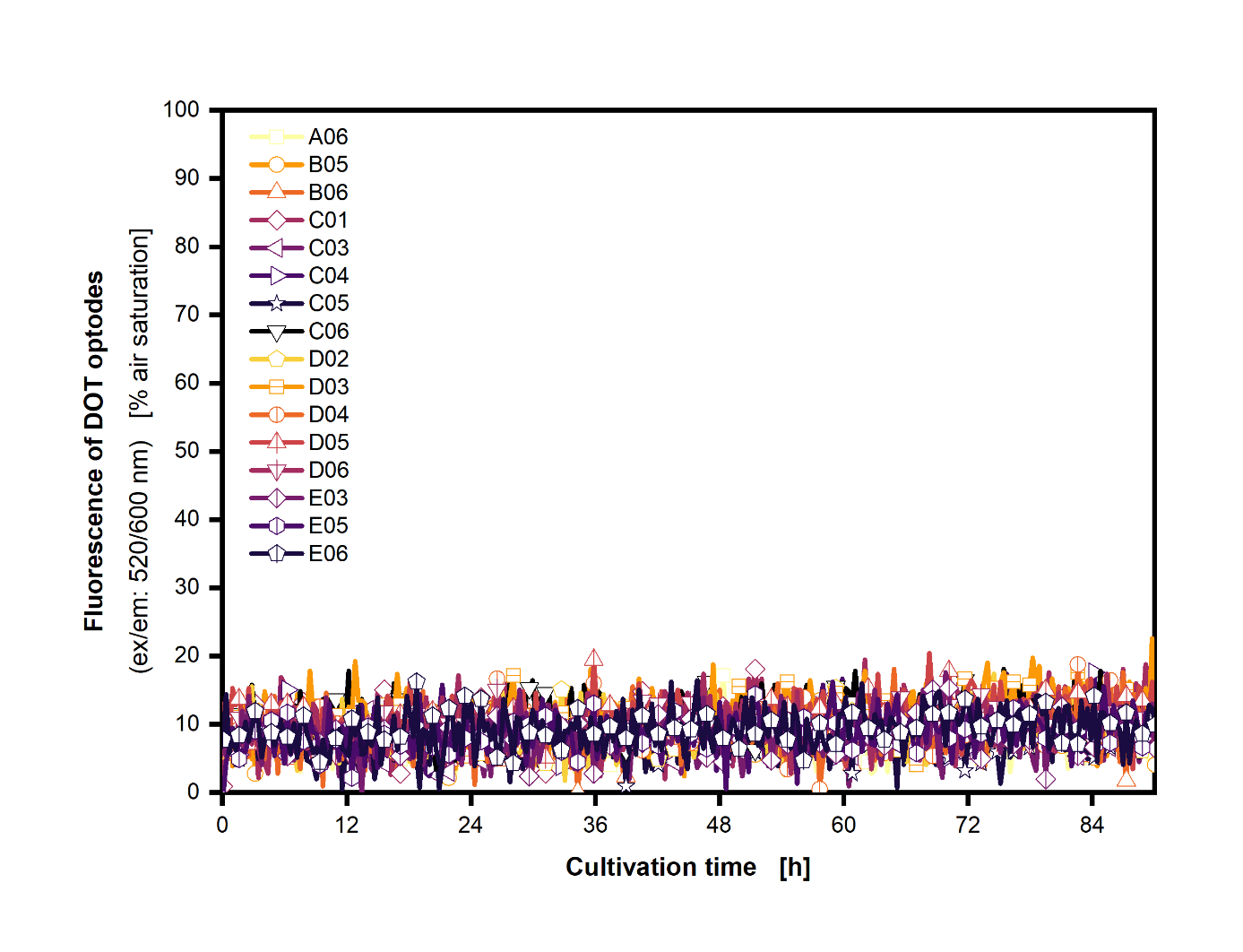
**

**Fig. S1 Oxygen measurement during *P. vulgatus* cultivation in a BioLector device** Online data of fluorescence of DOT optodes in different wells of one microtiter plate, ranging from 0 (no oxygen) to 100 (air saturation). DOT optode properties: precision: ± 5% dissolved oxygen, cross-sensitivity to complex media. Experimental setup corresponding to these results is graphically illustrated in Figure 1. The different well positions are designated in the legend. Medium = BHI (complex medium), c_buffer_ = 50 mM MOPS, c_glucose_ = 2.7 g L^-1^, T = 37 °C, n = 600 rpm, V_L_ = 2 mL, initial OD_600 nm_ = 0.13, initial pH after inoculation = 7.17-7.36, gas mix = 2% H_2_, 7% CO_2_ and 91% N_2_, N = 16, m2p labs BioLector

**
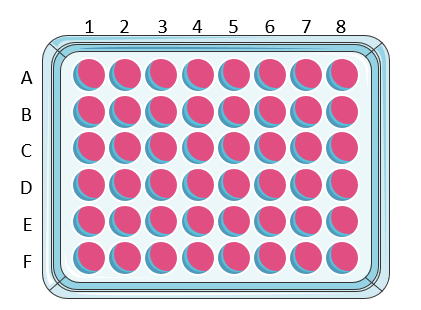
** **Fig. S2 Cultivation of *P. vulgatus* with online data in a BioLector device** (**a**) Scattered light, (**b**) NADH fluorescence, (**c**) Riboflavin fluorescence intensity. For clarity, only every 10^th^ measuring point is shown as a symbol. Well positions of the single cultivations are indicated in brackets (Fig. S2**c**). Well positions can be found in (**d**). Number of single wells for online monitoring are decreasing throughout the cultivation, due to sampling from those wells. Results shown here correspond to the results shown in Figure 2. 48-round-well microtiter plate, medium = DMMG, c_Glucose_ = 8 g L^-1^, c_buffer_ = 100 mM MOPS, T = 37 °C, n = 600 rpm, V_L_ = 2 mL, gas mix = 2% H_2_, 7% CO_2_ and 91% N_2_, N = 2-7, Parts of the figure were drawn by using pictures from Servier Medical Art. Servier Medical Art by Servier is licensed under a Creative Commons Attribution 3.0 Unported License. (https://creativecommons.org/licenses/by/3.0/)

**d**


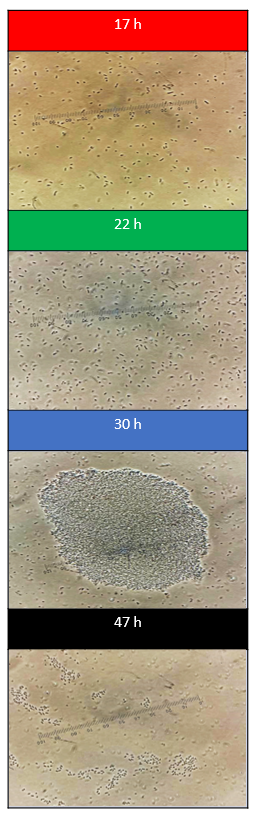


**Fig. S3 Microscopic pictures of cultivations of *P. vulgatus* in a BioLector device** 48-round-well microtiter plate, medium = DMMG, c_Glucose_ = 8 g L^-1^, c_buffer_ = 100 mM MOPS, T = 37 °C, n = 600 rpm, V_L_ = 2 mL, gas mix = 2% H_2_, 7% CO_2_ and 91% N_2_, N = 2-7. Samples were taken after 17, 22, 30 and 47 h of cultivation corresponding to the results shown in Figure 2a


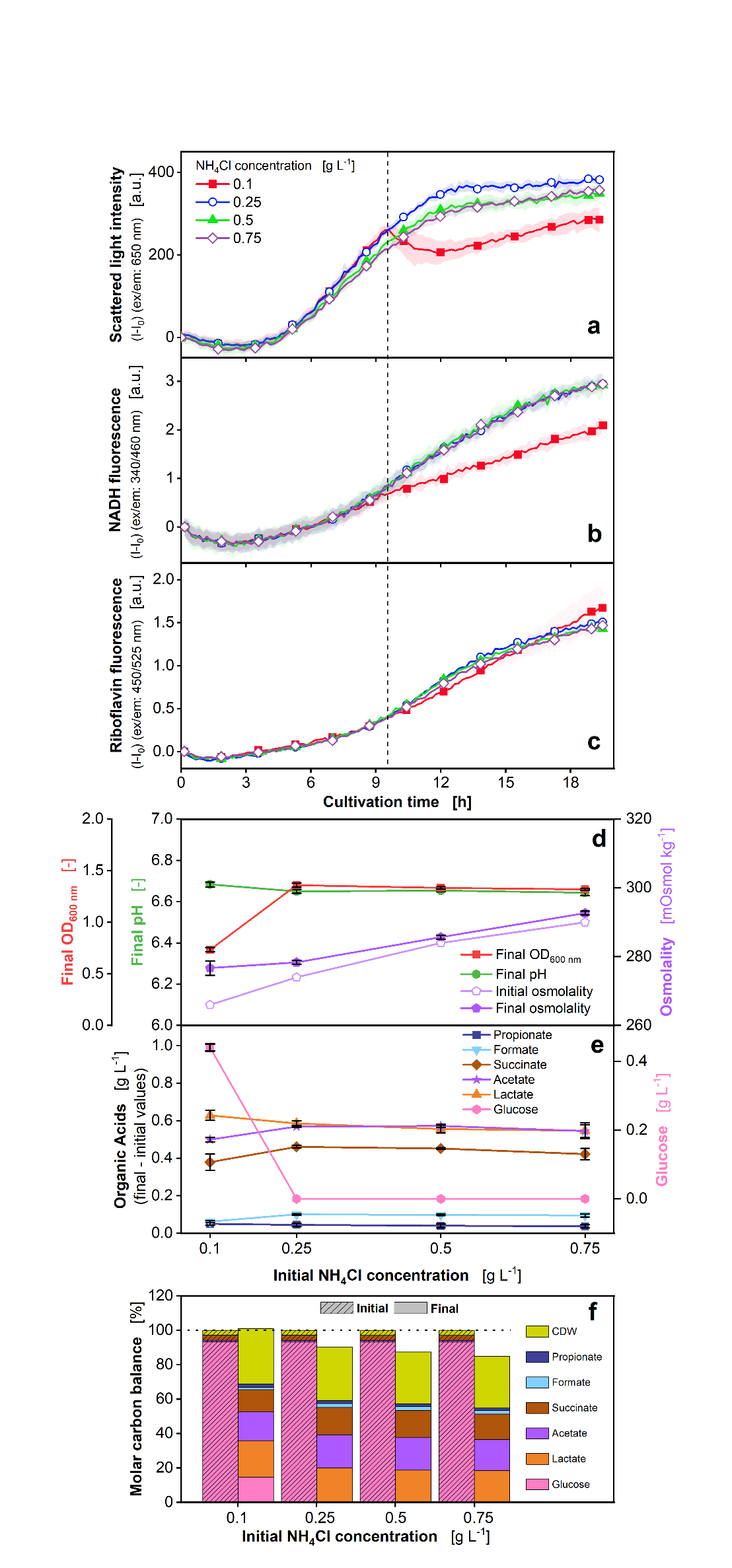


**Fig. S4 Effect of different NH_4_Cl concentrations on *P. vulgatus* in a BioLector device** Online data of (**a**) Scattered light, (**b**) NADH, and (**c**) Riboflavin fluorescence intensity. For clarity, only every 10^th^ measuring point is shown as a symbol. Shadows indicate standard deviations of four biological replicates. Due to high measurement accuracy, the shadows are partially barely visible. Vertical dashed line in (a) – (c) indicates nitrogen limitation of cultivation with 0.1 g L^-1^ NH_4_Cl. Offline data of four biological replicates of (**d**) Final OD_600 nm_, final pH and osmolality; (**e**) Produced organic acids including succinate, acetate, lactate, formate and propionate and glucose; (**f**) Carbon balance in % over the NH_4_Cl concentrations. The start of the fermentation was set to 100 %. 48-round-well microtiter plate, medium = DMMG, c_Glucose_ = 2.7 g L^-1^, c_buffer_ = 100 mM MOPS, T = 37 °C, n = 600 rpm, V_L_ = 2 mL, initial OD_600 nm_ = 0.15, initial pH after inoculation = 7.1-7.2, gas mix = 2% H_2_, 7% CO_2_ and 91% N_2_


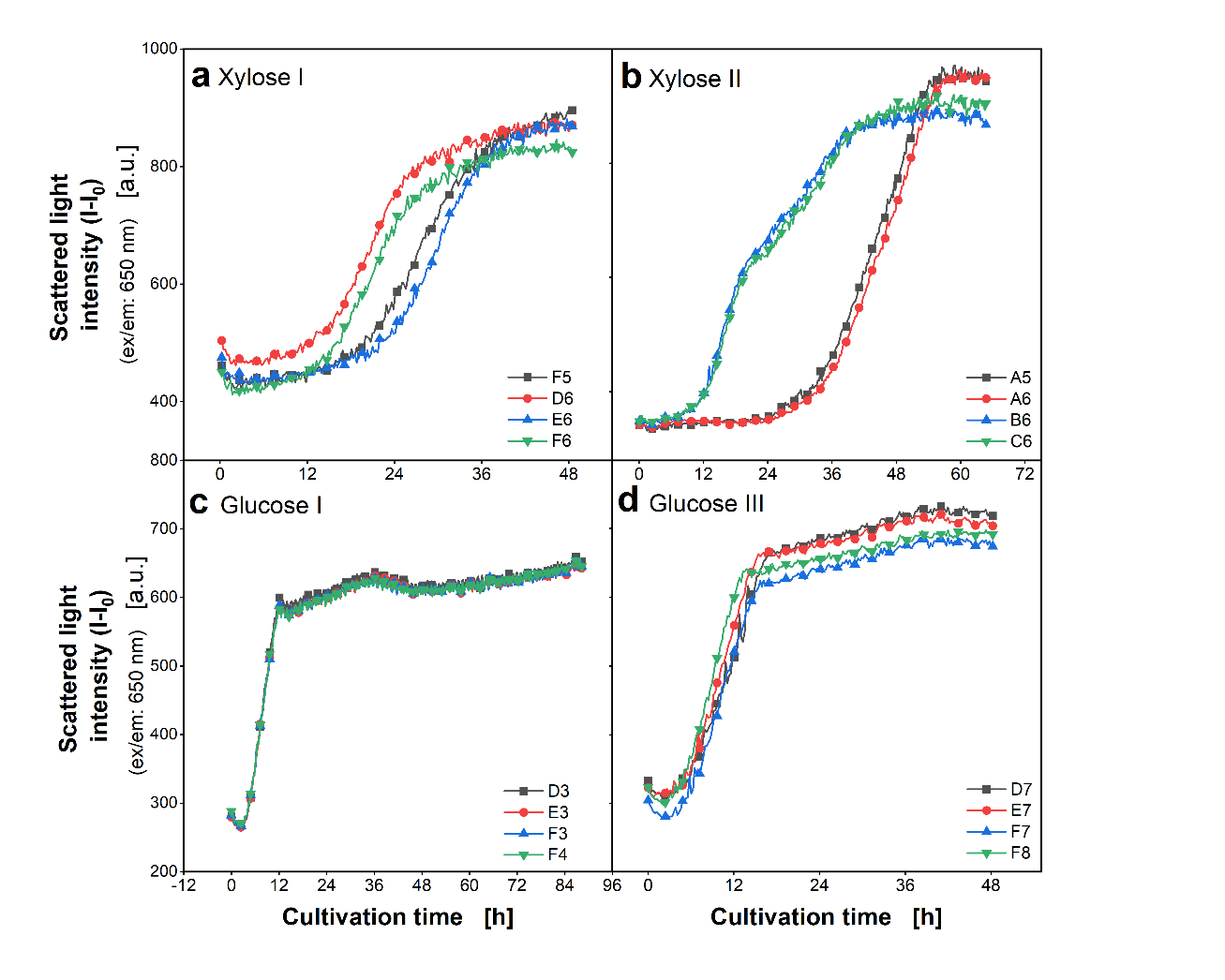

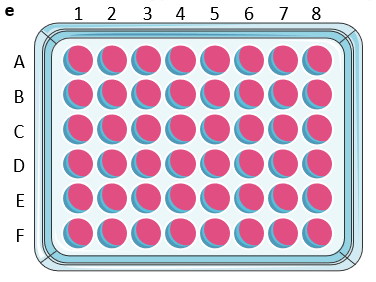


**e**

**Fig. S5 Variability of lag phase of *P. vulgatus* in a BioLector device with xylose, in comparison to glucose** Online data of four biological replicates each of scattered light intensity, (**a**) Xylose experiment I, (**b**) Xylose experiment II, (**c**) Glucose experiment I, (**d**) Glucose experiment III. Data was obtained from three different experiments in a period of 10 days. For clarity, only every 10^th^ measuring point is shown. Well positions of single cultivations are indicated in the legends. Well positions on microtiter plate can be found in (**e**). Results shown here correspond to the results shown in Figure 6. 48-round-well microtiter plate, medium = DMMG, c_Glucose_ = 2.7 g L^-1^ and molar carbon equivalents for other carbon sources, c_buffer_ = 100 mM MOPS, T = 37 °C, n = 600 rpm, V_L_ = 2 mL, initial OD_600 nm_ = 0.13, initial pH after inoculation = 7.0-7.1, gas mix = 2% H_2_, 7% CO_2_ and 91% N_2_, Parts of the figure were drawn by using pictures from Servier Medical Art. Servier Medical Art by Servier is licensed under a Creative Commons Attribution 3.0 Unported License. (https://creativecommons.org/licenses/by/3.0/)

**Fig. S6 Effect of different nitrogen sources on *P. vulgatus* in a BioLector device with standard deviations** Online average data of three biological replicates of (**a**) Scattered light, (**b**) NADH and (**c**) Riboflavin fluorescence intensity. Shadows indicate standard deviations of three biological replicates. For clarity, only every 24^th^ measuring point is shown as a symbol. Results shown here correspond to the results shown in Figure 7. 48-round-well microtiter plate, medium = DMMG, c_Glucose_ = 2.7 g L^-1^, c_buffer_ = 100 mM MOPS, 0.014 mol L^-1^ N_2_, T = 37 °C, n = 600 rpm, V_L_ = 2 mL, initial OD_600 nm_ = 0.13, initial pH after inoculation = 7.11-7.23, gas mix = 2% H_2_, 7% CO_2_ and 91% N_2_
